# Improving Detection of Hospital Adverse Events Using Machine Learning on Real-World Narrative EMR Data

**DOI:** 10.23889/ijpds.v9i5.2630

**Published:** 2024-09-18

**Authors:** Cheligeer Cheligeer, Guosong Wu, Seungwon Lee, Jie Pan 1, Natalie Sapiro, Danielle A. Southern, Cathy A. Eastwood, Hude Quan, Yuan Xu

**Affiliations:** 1 Centre for Health Informatics, Cumming School of Medicine, University of Calgary; 2 Provincial Research Data Services, Alberta Health Services; 3 Department of Community Health Sciences, Cumming School of Medicine, University of Calgary; 4 Lee Centre for Health Informatics, Cumming School of Medicine, University of Calgary; 5 Libin Cardiovascular Institute, University of Calgary; 6 Department of Oncology, Cumming School of Medicine, University of Calgary

## Objective

Administrative data often underrepresents hospital adverse events (AEs) due to limitations in International Classification of Diseases (ICD) coding. By leveraging electronic medical records (EMRs), we aim to mitigate these discrepancies and enhance the precision of healthcare surveillance and performance evaluations. To this end, we have developed a machine learning (ML)-based approach that utilizes EMR text data to detect common AEs.

## Methods

We sampled adult admissions from four Calgary hospitals (2017 - 2022). Registered nurses assessed charts for 17 AEs, and the results were used as reference standard. We compared two AE detection methods: the standard ICD-based method following Canadian guidelines, and our ML algorithm applied to EMR narratives. Sensitivity, positive predictive value (PPV), negative predictive value (NPV), and specificity for both methods were calculated and compared against the reference standard.

## Results

We analyzed 9,566 patients, of whom 1,506 were identified with AEs. Of the 17 AEs, the sensitivity in ICD-coded data ranged from 0-37%, and in EMRs, it was between 75-100%. Both showed low PPV (0-50% ICD vs.1-34% EMR). ICD data had high specificity ranging from 99-100% and NPV (99%-100%), while EMRs had specificity between 68-94% and an NPV of 100%.

## Conclusion and Implications

ML significantly enhances sensitivity for AE detection compared to ICD-10-CA coding, despite both methods experiencing low PPV due to imbalances in EMR data. This marked improvement in sensitivity highlights ML's potential to transform AE surveillance and reporting, promising significant advancements in patient safety and healthcare quality by enabling more accurate and comprehensive identification of AEs.

